# Venous thromboembolic disease in adults admitted to hospital in a setting with a high burden of HIV and TB

**DOI:** 10.7196/AJTCCM.2021.v27i3.155

**Published:** 2021-10-04

**Authors:** P Moodley, N A Martinson, W Joyimbana, K N Otwombe, P Abraham, K Motlhaoleng, V A Naidoo, E Variava

**Affiliations:** 1 Department of Internal Medicine, Faculty of Health Sciences, University of the Witwatersrand, Johannesburg, South Africa; 2 Perinatal HIV Research Unit, SAMRC Soweto Matlosana Collaborating Centre for HIV/AIDS and TB, University of the Witwatersrand, Johannesburg, South Africa; 3 NRF/DST Centre of Excellence in Biomedical TB Research, Johannesburg, South Africa; 4 Center for TB Research, Johns Hopkins University Baltimore, USA; 5 Department of Internal Medicine, Klerksdorp Tshepong Hospital Complex, South Africa

**Keywords:** deep vein thrombosis, pulmonary embolism, venous thromboembolism, prevalence, tuberculosis, HIV

## Abstract

**Background:**

HIV and tuberculosis (TB) independently cause an increased risk for venous thromboembolic disease (VTE): deep vein thrombosis (DVT) and/or pulmonary embolism (PE). Data from high HIV and TB burden settings describing VTE are scarce. The Wells’ DVT and PE scores are widely used but their utility in these settings has not been reported on extensively.

**Objectives:**

To evaluate new onset VTE, compare clinical characteristics by HIV status, and the presence or absence of TB disease in our setting. We also calculate the Wells’ score for all patients.

**Methods:**

A prospective cohort of adult in-patients with radiologically confirmed VTE were recruited into the study between September 2015 and May 2016. Demographics, presence of TB, HIV status, duration of treatment, CD4 count, viral load, VTE risk factors, and parameters to calculate the Wells’ score were collected.

**Results:**

We recruited 100 patients. Most of the patients were HIV-infected (*n*=59), 39 had TB disease and 32 were HIV/TB co-infected. Most of the patients had DVT only (*n*=83); 11 had PE, and 6 had both DVT and PE. More than a third of patients on antiretroviral treatment (ART) (43%; *n*=18/42) were on treatment for <6 months. Half of the patients (51%; *n*=20/39) were on TB treatment for <1 month. The median (interquartile range (IQR)) DVT and PE Wells’ score in all sub-groups was 3.0 (1.0 - 4.0) and 3.0 (2.5 - 4.5), respectively.

**Conclusion:**

HIV/TB co-infection appears to confer a risk for VTE, especially early after initiation of ART and/or TB treatment, and therefore requires careful monitoring for VTE and early initiation of thrombo-prophylaxis.

## Background

Venous thromboembolic disease (VTE) in the form of deep vein thrombosis (DVT) and pulmonary embolism (PE), is estimated to affect 1/10 000 Americans annually,^[1]^ and ~200 000 South Africans are estimated to present with DVT each year.^[2]^ VTE is associated with significant morbidity and mortality following diagnosis. The risk for VTE is increased with associated comorbidities.^[1]^

HIV is a risk factor for VTE, and some studies have suggested that it increases the risk of developing VTE up to tenfold compared with seronegative individuals.^[2,3]^ There are an estimated 7.5 million people living with HIV in South Africa (SA) and ~5 million are receiving antiretroviral therapy (ART).^[4]^ In people living with HIV, protein C and S deficiencies, raised circulating pro-inflammatory markers,^[3,5]^ and endothelial dysfunction ^[3,5-10]^ are risk factors for VTE. Furthermore, treatment with protease inhibitors and opportunistic infections are postulated to confer an increased risk.^[5,11-13]^ Patients on ART live longer, increasing the pool of individuals at risk for VTE.^[11]^

The World Health Organization (WHO) reported that the annual incidence of tuberculosis (TB) in SA was 520/100 000 population in 2018.^[14]^ Tuberculosis is an independent risk factor for VTE. Increased fibrinogen, factor VIII, plasminogen activator I and decreased anti-thrombin contribute to this risk.^[15]^ Severe TB involves disrupted fibrinolysis, decreased anti-thrombin III and thrombocytosis, which promotes a hypercoagulable state.^[16]^ Rifampin and isoniazid appear to accelerate this response.^[16]^ Moreover, rifampin causes dysregulation of coagulant factors and increases anticoagulant clearance.^[17]^ In SA, over 60% of TB patients are co-infected with HIV,^[14]^ and most of them are co-treated for both diseases.^[18,19]^ In high HIV and TB burden settings, therapy for VTE is often complicated by drug-drug interactions between treatment for VTE, TB and HIV.

Traditional risk factors associated with VTE include obesity,^[20]^ smoking,^[20]^ malignancy,^[20]^ prolonged travel (>6 hours),^[21]^ use of contraception,^[22]^ pregnancy and up to 28 days post-partum,^[23]^ prolonged immobility, recent major surgery, and paraparesis or orthopaedic cast of a limb.^[20]^ The Wells’ pre-test probability score for DVT^[24,25]^ and PE^[26,27]^ is used to estimate the probability of a PE or DVT. The presence of clinical parameters contributes to a composite score; low scores imply a low probability ^[28]^ and high scores imply an increased probability for VTE. However, few studies have reported Wells’ scores in patients from sub-Saharan Africa. Evidence or history of either HIV or TB disease is not part of the Wells’ scoring system. We therefore prospectively evaluated new-onset VTE in our setting of high HIV/TB co-infection, comparing clinical characteristics by HIV status, and the presence or absence of TB disease, and calculated the Wells’ scores in all patients.

## Methods

Ethical approval was granted by the Human Research Ethics Committee (Medical) of the University of the Witwatersrand, Johannesburg (ref. no. M15740).

A cohort of adult in-patients diagnosed with VTE from September 2015 to May 2016 were recruited prospectively at Tshepong Hospital, North West Province. All patients aged 18 years and older with a radiologically confirmed DVT or PE were approached. DVT was confirmed by Doppler ultrasound of the affected limb illustrating at least one of the following: presence of a thrombus; or noncompressibility or diminished venous flow^[2]^ conducted on patients with signs and symptoms of suspected DVT. PE was confirmed by computed tomography (CT) pulmonary angiogram showing a filling defect(s) or embolus seen in the pulmonary artery/arteries following presentation with signs and symptoms of PE.^[29]^ Radiology records were reviewed daily and those diagnosed with VTE were approached. Written informed consent was obtained.

Patients were interviewed, and clinical and laboratory data were obtained from routine hospital records – all in-patients are offered HIV testing routinely following pre-test counselling. Interviews involved documentation of risk factors for VTE. In all patients, smoking and travel history (prolonged air or road travel >6 hours) within the last four weeks was obtained. History of injectable or oral hormonal contraceptive use within the last 3 months, and current and recent pregnancy were recorded. Patient’s HIV status, ART details including onset and duration, any defaulting period (longer than 2 months), as well as most recent viral load (VL) and CD4 cell count were documented. Diagnosis of active TB, site, initiation of treatment, duration and type were noted. Weight and height were used to calculate body mass index (BMI). Wells’ score for PE and DVT was calculated for each patient. When assessing probability of a DVT, a score of 3 - 8 points suggests high probability, 1 - 2 moderate probability and <2 low probability.^[24,25]^ Regarding the Wells’ score for probability of PE, a total <2 suggests low probability, a score ≤2 and ≤6 moderate probability and a score >6 implies high probability.^[26,27]^ To calculate the Wells’ score for DVT, collateral non-varicose veins, localised tenderness, swelling and/or pitting oedema of the entire affected limb and calf swelling more than 3 cm in the symptomatic limb were noted.^[24]^ In those with a PE, the electrocardiogram (ECG) was analysed for features of sinus tachycardia and right ventricular strain: S_1_Q_3_T_3_ pattern and tall R-wave in standard lead V_1_ with non-specific T-wave changes in anterior leads.^[30]^

### Statistical analysis

Categorical variables and continuous variables were reported, stratified by overall HIV status and by the presence or absence of TB disease. χ² or Fisher’s exact test was used to determine the association between categorical variables, and t-test or Kruskal-Wallis test was used to compare continuous variables. *P*-values were obtained for all variables considered. Variables with *p*-values ≤0.05 were considered as significant. Analysis was performed using SAS Enterprise Guide 7.1 (SAS, USA). Study data were collected and managed using research electronic data capture (REDCap) hosted at the University of the Witwatersrand.^[31,32]^

## Results

A total of 125 adult patients were diagnosed with VTE at Tshepong Hospital over the study period, and 80% (*n*=100) of them consented to participate in the present study. These patients represent 1.5% of all adult medical admissions (*n*=6 777) to the hospital over the study period. Those not recruited had either been treated as outpatients or were referred to other hospitals.

DVT was diagnosed in 83 patients, PE in 11 patients and 6 had both a PE and DVT. In the 89 patients with a DVT, 94.4% (*n*=84) had unilateral limb DVT, while 13.5% (*n*=12) had bilateral lower limb DVT. Of the 17 patients with a PE, 64.7% (*n*=11) had bilateral lung involvement. Most of the patients were women (*n*=67). Overall, the median (interquartile range (IQR)) age was 47.0 (35.0 - 57.0) years. The median (IQR) BMI was 23.3 (19.9 - 31.0) for 96 patients from whom the measurements were obtainable (Table 1). The median (IQR) Wells’ score for DVT patients was 3.0 (1.0 - 4.0). The median (IQR) Wells’ score for PE patients was 3.0 (2.5 - 4.5). Sinus tachycardia was the most common ECG finding in PE patients (47.0%; *n*=8). Nine patients died during hospitalisation after a median (IQR) of 25.0 (20.0 - 31.0) days after admission, and 3 of them were women. All patients who died had been diagnosed with DVT only. The median (IQR) duration of admission for discharged patients was 12.5 (6.0 - 18.0) days.

### HIV and VTE

Fifty-nine patients were HIV-positive, their median (IQR) age was 40.0 (32.5 - 50.0) years, and more than two-thirds of them (69.5%; *n*=41) were women. Of the HIV-positive patients, 89.8% (*n*=53) were diagnosed with DVT, 6.8% (*n*=4) with a PE and 3.4% (*n*=2) had both. VL was available for 64.4% (*n*=38) patients: 28.9% (*n*=11) were virally suppressed (<50 copies/mL); 52.6% (*n*=20) had a VL of 50 - 1 000 copies/mL; and 47.3% (*n*=18) had a VL >1 000 copies/mL. Almost all patients (96.0%; *n*=57) had CD4 cell count results and their median (IQR) CD4 cell count was 130.0 (58.0 - 351.0) cells/µL. Thirty-four patients (59.7%) had a CD4 cell count <200 cells/µL and 26 of these patients were co-infected with HIV and TB. Those who were HIV-positive without TB had a higher median (IQR) CD4 cell count of 352.0 (42.0 - 451.0) cells/µL than those with TB (*p*=<0.0001) (Table 1).

**Table 1:** Overall summary of demographics, diagnosis, and clinical and laboratory findings stratified by HIV and TB infection

		**HIV-positive**		**HIV-seronegative**	
	**Overall**	**TB disease**	**No TB**	**TB disease**	**No TB**
	**(N=100),**	**(n=32),**	**(n=27),**	**(n=7),**	**(n=34),**
**Characteristics**	n(%)*	n(%)*	n(%)*	n(%)*	n(%)*
**Age (years), median (IQR)**	47.0 (35.0 - 57.0)	39.0 (32.0 - 43.5)	44.0 (35.0 - 59.0)	53.0 (31.0 - 60.5)	56.0 (48.0 - 65.0)
**Gender**					
Female	67 (67.0)	22 (68.8)	19 (70.4)	4 (57.1)	22 (64.7)
**BMI, median (IQR)**	23.3 (20.0 - 31.1)	20.1 (17.0 - 22.9)	24.1 (21.2 - 32.0)	21.6 (21.1 - 23.4)	30.7 (23.3 - 38.2)
Obese	27/96 (28.1)	0/30 (0.0)	10/26 (38.5)	0/7 (0.0)	17/33 (51.5)
**Diagnosis**					
DVT	83 (83.0)	30 (93.8)	23 (85.2)	4 (57.1)	26 (76.5)
PE	11 (11.0)	1 (3.1)	3 (11.1)	2 (28.6)	5 (14.7)
DVT and PE	6 (6.0)	1 (3.1)	1 (3.7)	1 (14.3)	3 (8.8)
**Wells’ score (DVT)**	n=89	n=31	n=24	n=5	n=29
Moderate risk	23 (25.8)	9 (29.0)	7 (29.2)	1 (20.0)	6 (20.7)
High risk	64 (71.9)	22 (80.0)	17 (70.8)	4 (80.0)	21 (72.4)
Median	3 (1.0 - 4.0)	3 (1.0 - 3.0)	3.0 (1.0 - 4.0)	3 (2.0 - 3.0)	3 (1.5 - 4.0)
**Wells’ score (PE)**	n=17	n=2	n=4	n=3	n=8
Moderate risk	9 (52.9)	1 (50.0)	2 (50.0)	1 (33.3)	5 (62.5)
High risk	3 (17.7)	1 (50.0)	1 (25.0)	0 (0.0)	1 (12.5)
Median (IQR)	3 (2.5 - 4.5)	5.25 (3.0 - 7.5)	3.8 (2.3 - 5.8)	1.5 (1.5 - 4.5)	3 (3 - 4.5)
**CD4 cell count (cells/µL), median (IQR)**	130.0 (58.0 - 351.0)	75.5 (38.0 - 135.0)	352.0 (142.0 - 451.0)	-	-
**≤200**	34(59.7)	26/32 (81.3)	8/25 (32.0)†	-	-
**>200**	23 (40.35)	6/32 (18.8)	17/25 (68.0)†	-	-
**Viral load (copies/mL), median (IQR)**	968.5 (0.0 - 128 961.3)	106 564.0 (250.5 - 431 016.0)	51 (0.0 - 1881.0)	-	-
**Viral suppression**	11/38‡	2/19	9/19		

IQR = interquartile range

BMI = body mass index

DVT = deep vein thrombosis

PE = pulmonary embolism

* Unless otherwise specified

^†^ Only 25 out of 27 CD4 counts available

^‡^ Only 38 viral load results available

Three-quarters of HIV-positive patients (74.6%; *n*=44) had been initiated on ART prior to VTE diagnosis and one after diagnosis. A single patient was unsure of timing of initiating ART. The median (IQR) duration on ART was 327.0 (60.0 - 1 601.5) days (Table 2).

**Table 2:** Factors related to HIV treatment and TB treatment according to HIV-positive and HIV-negative subgroups

		**HIV-positive**		**HIV-seronegative**	
	**Overall**	**TB disease**	**No TB**	**TB disease**	**No TB**
**Characteristics**	median (IQR)*	median (IQR)*	median (IQR)*	median (IQR)*	median (IQR)*
**ART therapy, n (%)**	45 (76.3)	25 (78.1)	20 (74.1)	-	-
**Time on ART therapy (days)**	327.0 (60.0 - 1601.5)	129.5 (39.5 - 716.0)	1023.5 (197.5 - 2684.0)	-	-
**TB treatment, *n***	39	32	-	7	-
**Time on TB treatment (days)**	27.0 (5.0 - 62.0)	40.5 (7.0 - 70.0)	-	6 .0 (2.0 - 13.0)	-

IQR = interquartile range

ART = antiretroviral therapy

TB = tuberculosis

* Unless otherwise specified

**Fig. 1:**
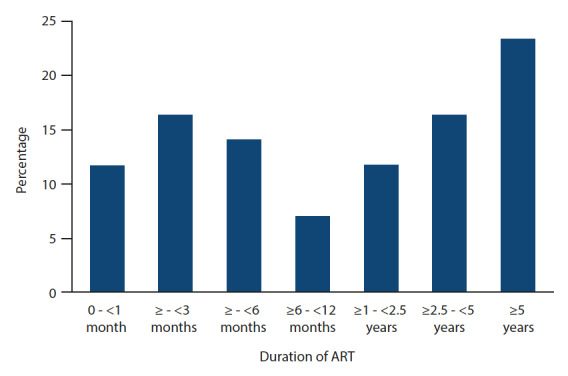
Patients grouped according to the duration of ART prior to onset of VTE (n=43) ART = antiretroviral therapy VTE = venous thromboembolism

Two-fifths of patients (40.9%; *n*=18) had started ART within 6 months (Fig. 1), with 14 of this group having TB co-infection. Most patients were receiving a fixed dose combination (FDC) of tenofovir, efavirenz and emtricitabine.^[18]^ Only four patients were receiving protease inhibitors.

Of the HIV-seronegative patients, 63.4% (*n*=26) were women. Thirty seronegative patients had a DVT, 7 had PE and 4 had both DVT and PE. Patients who were HIV-negative were older than seropositive patients with a median (IQR) age of 56.0 (47.0 - 64.0) years v. 40.0 (32.0 - 51.0) years (*p*=<0.0001).

### Tuberculosis

Overall, 39 out of 100 VTE patients had TB. TB was laboratory confirmed in 24 patients and 29 had radiological evidence of pulmonary TB. Most patients (82.0%; *n*=32) were co-infected with HIV. The HIV/TB co-infected patients had a median (IQR) age of 39.0 (32.0 - 43.5) years compared with those with TB infection alone at 53.0 (31.0 - 60.5) years (*p*=0.35).

The median (IQR) CD4 cell count for HIV/TB co-infected patients was 75.5 cells/µL (38.0 - 135.0) with a median VL of 106 564.0 copies/mL (250.5 - 431 016.0). Twenty-five patients were on ART and only 2 were virally suppressed (Table 1).

**Fig. 2:**
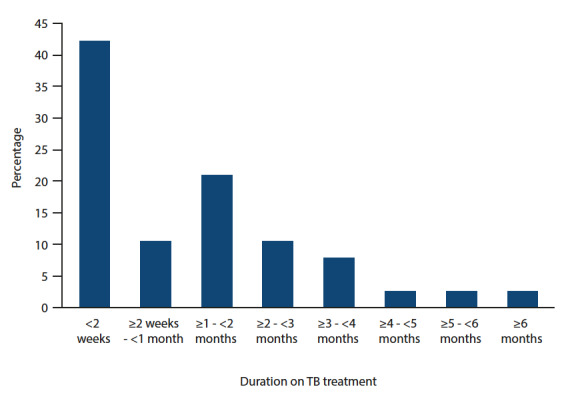
Patients grouped according to the duration of TB treatment prior to onset of VTE (n=38) ART = antiretroviral therapy VTE = venous thromboembolism

Thirty-eight patients were already on TB treatment prior to VTE diagnosis (one patient started after diagnosis). The median (IQR) duration on TB treatment was 27.0 (5.0 - 62.0) days (Table 2). Venous thromboembolism was diagnosed in 52.6% (*n*=20) of TB patients within the first month of initiating rifampicin-based TB treatment and of these, 42% (*n*=16) within 2 weeks of initiating TB treatment (Fig. 2). Of this group of 20 patients, 6 were HIV-negative. Most of the HIV/TB co-infected patients (*n*=10/14) were on ART, and 5 of them were on ART for <6 months. More than three-quarters of patients (76.3%; *n*=29) were in the intensive phase of TB treatment.^[19]^ Four patients were receiving treatment for drug-resistant TB. Over the study period, 18.2% (*n*=1 236) of adults admitted to the adult medical wards at Tshepong Hospital had a diagnosis of TB.

### Wells’ score

All sub-groups of patients with a DVT had a median (IQR) Wells’ score of 3.0 (1.0 - 4.0) (Table 1). Pitting oedema in the affected leg (71.7%), localised calf tenderness (56.6%) and calf swelling more than 3 cm (48.5%) were the most common parameters seen in all patients with DVT. However, in the HIV-positive group (TB included), pitting oedema was observed in 68.5% of the patients, 53.7 had calf swelling more than 3 cm and, 22.2% had collateral non-varicose superficial veins.

The median (IQR) Wells’ score for all patients diagnosed with PE was 3.0 (2.5 - 4.5). The HIV-positive only and HIV/TB co-infected group had the highest median (IQR) Wells’ scores of 3.8 (3.0. - 7.0) and 5.3 (3.0 - 7.5), respectively (Table 1).

### Traditional risk factors

**Fig. 3:**
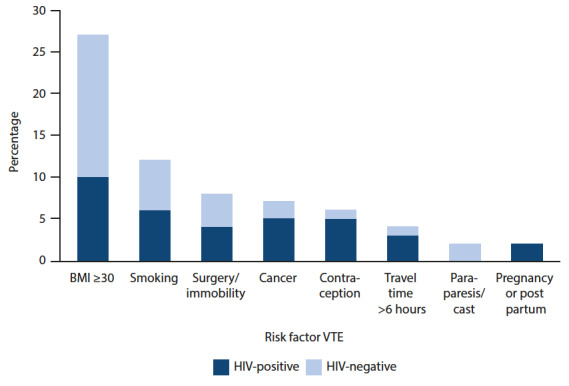
Percentage of study population with traditional risk factors for VTE according to HIV status (n=100) VTE = venous thromboembolism

Thirty-six patients had a smoking history, and 4.0% of women and 8.0% of men self-reported smoking at the time of diagnosis of VTE (current smokers). Twenty-seven patients were obese (BMI >30 kg/m² ), of whom 10 were HIV-positive. Seven patients had a malignancy (5 had Kaposi sarcoma). Recent major surgery and/or immobilisation were reported by 8 patients, and 6 women were using contraception (Fig. 3).

## Discussion

There are few studies in sub-Saharan Africa reporting factors related to HIV and TB in patients with VTE. We found a high prevalence of HIV and TB among those with VTE, suggesting that these are strong risk factors for thromboembolic disease. Less than a tenth of our patients (9%) died at a median time of 25 days after admission, demonstrating the human and financial cost of this illness to the healthcare system.

The overall prevalence of VTE among adult patients admitted to the medical wards was 1.5% over the study period. Studies in developed countries report 2 - 10-fold increased risk of VTE in HIV-positive individualscompared with their HIV-negative counterparts.^[8,33]^ The majority of patients with VTE (59%) in our study were HIV-positive, as reported in other studies in SA.^[2,34]^ However, HIV prevalence in the present study was markedly higher than the general HIV prevalence (12.7%) in SA.^[4]^ Similarly, the prevalence of TB in our study population was higher (39%) than the prevalence reported in adults admitted over the study period (18.2%), and most TB patients were HIV co-infected. Studies in similar hospital settings have reported comparable prevalence of TB in those with DVT in SA.^[2,9]^ It has been estimated that 3 - 4% of patients with TB develop VTE, with the mortality of in-patients with combined VTE and active TB being greater than the risk of TB or VTE alone.^[35]^

Unsurprisingly, the median age of the HIV-positive patients with VTE was younger than the HIV-negative patients in our study. Young people aged between 15 and 34.9 years old have the highest prevalence of HIV in SA.^[4]^ Similarly to other SA studies, women comprised 67.0% of all patients in our present study.^[10,4]^ Studies carried out in developed settings show, in contrast to ours, a predominance of male patients with VTE,^[5,11]^ possibly reflecting different risks for HIV^[36]^ in our setting where the epidemic predominantly affects women.^[4,37]^ Severe immunodeficiency was a dominant finding among the HIV-positive group – most had CD4 counts <200 cells/µL, similar to other studies.^[3,9,29,36,38,39]^ Those co-infected with HIV and TB had markedly lower CD4 cell counts. Interestingly, VLs were not uniformly high, consistent with other studies.^[3,5,9,29]^

Two-fifths of patients (40%) in our study initiated ART within 6 months prior to VTE. Levels of markers of endothelial cell dysfunction and coagulation were found to be abnormal in HIV-positive patients recently initiated on combined ART therapy.^[40]^ Mjiluf-Cruz *et al*. ^[41]^ found the median time to onset of VTE following ART initiation to be 7 months, which suggests that immune reconstitution following ART initiation may be contributing to the onset of VTE. Immune reconstitution in the form of an increase in number of CD4 and CD8 T lymphocytes occurs in the first 3 - 6 months following ART initiation.^[42]^ This may lead to increased circulating pro-inflammatory markers and activation of the inflammatory cascade resulting in a prothrombotic state. However, others have not reported similar findings.^[5,43]^ In our present study, most of those who had recently initiated ART and developed VTE had TB co-infection. Of the 12 patients who were diagnosed with VTE within 3 months after initiating ART, 9 had TB, suggesting that TB and its treatment may exacerbate the thrombotic risk of VTE immune reconstitution syndrome following ART. Numerous studies have shown the correlation of protease inhibitor-containing regimens^[41,44,45]^ and the onset of VTE. Only 4 patients were on a PI-containing regimen in our present study.

Tuberculosis has been found to create a hypercoagulable state owing to various mechanisms.^[16,17,35,46,47]^ Anti-TB treatment also contributes to the risk for VTE, particularly 2 weeks after initiating rifampicin.^[17]^ Rifampin induces cytochrome (CYP) 3A4,^[48,49]^ which metabolises warfarin,^[48–51]^ leading to ineffective anticoagulation. Similar effects occur with non-nucleoside reverse transcriptase inhibitors and protease inhibitors.^[51-53]^ Isoniazid inhibits CYP P450, increasing the effects of warfarin.^[51]^ Newer anticoagulants such as dabigatran and rivaroxaban require less monitoring and are said to have fewer drug interactions in those receiving therapy for TB or HIV.^[54,55]^ Some studies have shown these agents to be efficacious and cost effective in developed countries.^[56]^ There are a few studies analysing the cost effectiveness of these newer agents in public hospitals in developing countries.^[57]^

Strikingly, most of the HIV-seronegative patients diagnosed with TB presented within 1 month of TB diagnosis, suggesting an immune reconstitution-related hypercoagulable state following the initiation of TB treatment.

Patients with a BMI >30 kg/m² were predominantly HIV-seronegative, suggesting that obesity may not be a major predisposing factor for VTE in HIV-infected adults.^[10]^ Only 6 patients had a ≥20 packs-a-year smoking history. Smoking has been shown to be a risk factor for VTE^[58,59]^ in conjunction with other risk factors such as HIV.^[5]^ Seven patients in our present study were diagnosed with a malignant process, 5 of whom had HIV-related Kaposi sarcoma (8.5% of HIV-positive group). Crum-Cianflone *et al*. ^[5]^ similarly found that 6.0% of HIV-positive adults with VTE had cancer.^[5]^ This differs from another SA study that reported malignancy to be high in HIV-negative patients.^[34]^ Kaposi’s sarcoma is related to VTE development owing to vessel compression and infiltration.^[38]^ The Wells’ scores for those with a DVT was the same in all the HIV and/or TB sub-groups. In each HIV/TB sub-group, scores suggested moderate to high probability for VTE, but HIV/TB co-infected patients did not appear to have a significantly higher Wells’ score for DVT. More research is needed to assess a modification to the Wells’ score that will incorporate HIV and TB disease status, and possibly duration of therapy.

### Study limitations

Several patients had missing clinical data. We did not include controls without VTE, making it difficult to assess the characteristics of Wells’ scores in HIV and HIV/TB co-infected patients. Measures of coagulation were not routinely done, and D-dimers were not measured in many patients. However, D-dimers are used for their negative predictive value, and all our cases were proven radiologically.

## Conclusion

Our study illustrates the apparent contribution that HIV, TB and their therapies confer on incident VTE, as well as a possible immune reconstitution-related hypercoagulable state soon after starting ART and/or anti-TB therapy. Further studies are warranted to assess whether thrombo-prophylaxis would counter the hypercoagulable state that may exist in HIV-positive patients with TB receiving rifampicin treatment.
